# Tissue-Protective Effects of NKG2A in Immune-Mediated Clearance of Virus Infection

**DOI:** 10.1371/journal.pone.0108385

**Published:** 2014-09-24

**Authors:** Kenneth H. Ely, Mitsuo Matsuoka, Matthew P. DeBerge, Jessica A. Ruby, Jun Liu, Mark J. Schneider, Yan Wang, Young S. Hahn, Richard I. Enelow

**Affiliations:** 1 Department of Medicine, Geisel School of Medicine at Dartmouth, Lebanon, New Hampshire, United States of America; 2 Department of Medicine, Yale University School of Medicine, New Haven, Connecticut, United States of America; 3 Beirne B. Carter Center for Immunology Research, University of Virginia School of Medicine, Charlottesville, Virginia, United States of America; 4 Department of Microbiology/Immunology, Geisel School of Medicine at Dartmouth, Lebanon, New Hampshire, United States of America; University of Missouri-Columbia, United States of America

## Abstract

Virus infection triggers a CD8^+^ T cell response that aids in virus clearance, but also expresses effector functions that may result in tissue injury. CD8^+^ T cells express a variety of activating and inhibiting ligands, though regulation of the expression of inhibitory receptors is not well understood. The ligand for the inhibitory receptor, NKG2A, is the non-classical MHC-I molecule Qa1^b^, which may also serve as a putative restricting element for the T cell receptors of purported regulatory CD8^+^ T cells. We have previously shown that Qa1^b^-null mice suffer considerably enhanced immunopathologic lung injury in the context of CD8^+^ T cell-mediated clearance of influenza infection, as well as evidence in a non-viral system that failure to ligate NKG2A on CD8^+^ effector T cells may represent an important component of this process. In this report, we examine the requirements for induction of NKG2A expression, and show that NKG2A expression by CD8^+^ T cells occurs as a result of migration from the MLN to the inflammatory lung environment, irrespective of peripheral antigen recognition. Further, we confirmed that NKG2A is a mediator in limiting immunopathology in virus infection using mice with a targeted deletion of NKG2A, and infecting the mutants with two different viruses, influenza and adenovirus. In neither infection is virus clearance altered. In influenza infection, the enhanced lung injury was associated with increased chemoattractant production, increased infiltration of inflammatory cells, and significantly enhanced alveolar hemorrhage. The primary mechanism of enhanced injury was the loss of negative regulation of CD8^+^ T cell effector function. A similar effect was observed in the livers of mutant mice infected intravenously with adenovirus. These results demonstrate the immunoregulatory role of CD8^+^ NKG2A expression in virus infection, which negatively regulates T cell effector functions and contributes to protection of tissue integrity during virus clearance.

## Introduction

Acute virus infection results in induction of innate immune responses, which serve to contain replication as well as enhance activation of adaptive responses. The adaptive responses are critical to clearance of the virus, though at potentially significant cost to the host. The cost to the host depends upon multiple factors, in particular the kinetics of virus replication (and hence antigen load) relative to the kinetics of T cell activation, acquisition of effector activity, and magnitude of the response. Disproportionate kinetics may lead to a potent T cell response encountering a large antigen load resulting in significant tissue destruction [1], [2]. The consequences to the host also depend to a tremendous extent on the particular tissue involved in the infection and clearance. The lung is an example of an organ to which considerable injury is unlikely to be compatible with host survival, depending upon the specific components of the respiratory system involved. An overwhelming infection of large airways, coupled with a robust T cell response, would likely result in considerable morbidity (as in the 1957 influenza pandemic), and significant susceptibility to secondary bacterial pneumonia (resulting from multiple potential mechanisms currently under investigation [3]–[7]). In contrast, the significant mortality attributed to infection with avian strains (such as H5N1 [8]) and likely to extraordinary mortality observed in the 1918 pandemic (characterized by an unusual mortality distribution, and descriptions from the period, which include instances of very short intervals between first symptoms and death [9]) are likely attributable to multiple factors, including highly efficient replication [10], as well as viral tropism for the distal airways, i.e. the alveolar epithelium [10], [11]. Significant injury to alveolar epithelial cells may have an enormous impact on respiratory gas exchange, which is often incompatible with host survival [12]. CD8^+^ T cell effector mechanisms are particularly important contributors to the immunopathology observed in response to virus infection [13]–[15].

The initial activation of T cell responses is thought to involve multiple early events which are “programmed”, and though there are a number of early modifiers of the magnitude of the T cells [16]–[18], there are parallel mechanisms which are induced to counteract potentially deleterious effector functions, some of which are extrinsic to the effector CD8+ T cells, such as Tregs [19], [20], and some of which are intrinsic to the effector cells. Some intrinsic mechanisms include effector cell IL-10 production [21], as well as induced expression of surface molecules that deliver inhibitory signals upon ligation, such as CTLA-4, PD-1, Lag-3, CD160, and others [22]. One such inhibitory molecule whose expression is induced upon effector CD8^+^ T cells is NKG2A, which is expressed as a heterodimer with CD94 [23]–[25]. Though expressed constitutively on most NK cells, NKG2A is a product of the KLRC1 gene and is a member of a family of inhibitory NK receptors (iNKR), all originally described on NK cells but more recently found on other cell types, though primarily CD8^+^ T cells [24]–[29]. The interaction of iNKRs on CD8^+^ T cells with MHC class I ligands on APCs has been proposed to allow for fine-tuning TCR-mediated activation of CD8^+^ T cells [30], [31]. Among NKRs, the CD94/NKG2A heterodimer which recognizes the non-classic MHC class I molecules HLA-E in human and Qa-1^b^ in mouse, is conserved between human and mouse. Because of the short cytoplasmic tail on CD94, engagement of CD94/NKG2A heterodimer with its receptor transduces a negative signal through the two intracytoplasmic immunoreceptor tyrosine-based inhibitory motifs (ITIM) of NKG2A molecule [31]. We [28] and others [25], [32] have shown that CD94/NKG2A engagement on antiviral CD8^+^ T cells may be associated with dampened effector activity (e.g., cytotoxicity, cytokine production), and we have also shown induction of NKG2A expression on antigen specific CD8^+^ T cell following influenza infection in mice [28]. We further demonstrated that mice deficient in Qa1^b^ exhibit enhanced immunopathology upon CD8^+^ T cell-mediated clearance of influenza infection [28]. Additionally, studies in a non-infectious model of CD8^+^ T cell-mediated lung injury, antibody blockade of NKG2A enhanced lung pathology, in large part by augmenting the release of inflammatory cytokine TNF-α upon TCR engagement, which we have previously shown to be a critical mediator of pulmonary immunopathology [13], [14], [33]–[35].

An alternative role for the Qa1^b^ has been proposed as the restricting element for a novel CD8^+^ regulatory T cell [36], offering an alternative interpretation of the enhanced immunopathology we observed in influenza-infected Qa1^b−/−^ mice. In order to formally confirm that this phenotype was due, at least to a significant extent, to the absent engagement of NKG2A on effector CD8^+^ T cells, we generated mice with a targeted deletion of the NKG2A and observed their responses to virus infection. Upon infection of these mutants we observed enhanced tissue injury in immune-mediated clearance of two model virus infections, influenza and adenovirus, in the lung and the liver, respectively, supporting an important role for NKG2A in limiting effector CD8^+^ T cell function.

## Materials and Methods

### Ethics statement

All mice were treated humanely and all studies were conducted in accordance with guidelines approved by the Association for Assessment and Accreditation of Laboratory Animal Care (AALAC) using a protocol (enel.ri.1) approved by the Institutional Animal Care and Use Committee (IACUC) at the Geisel School of Medicine at Dartmouth. In accordance with our IACUC approved protocol, mice were euthanized via CO_2_ inhalation under anesthesia or anesthesia overdose and exsanguination. A composite endpoint was used as a surrogate for survival and mice were sacrificed, as above, either after losing ≥20% of the initial body weight, or other signs of severe illness, such as huddling and piloerection.

### Mice, viruses and infections

BALB/c mice 6–10 weeks old were purchase from Taconic (Germantown, NY), C57BL/6 mice 6–10 weeks old were purchased from the Jackson Laboratories (Bar Harbor, ME) and NKG2A^−/−^ mice were generated as described below. Mice were intranasally infected with 0.5LD_50_ influenza A/PR8/34 (H1N1) virus, a mouse adapted laboratory strain. Recombinant adenovirus expressing ovalbumin (Ad-OVA) was generously provided by Dr. Timothy L. Ratliff (University of Iowa) and the Iowa Gene Transfer Vector Core (Iowa City, IA). Mice were infected with 5×10^8^ PFU of rAd per mouse by IV immunization.

### Generation of NKG2A-deficient mice

129v/Sj ES cells (ATCC) were maintained on a layer of mitomycin C-treated MEFs, supplemented with LIF and 15% FBS. Briefly a clone containing the mouse NKG2A/KLRC1 gene (NCBI gene ID:16641) was isolated from a 129/J mouse genomic library. A targeting vector was designed to replace exons 1 and 2 (as well as part of exon 3) and was generated with a 1.63 kb short arm, and 4.68 kb long arm with a *neo* insert in between. The insert was flanked on the 5′ end with CMV promoter-driven thymidine kinase gene. The vector was linearized with *cla*1 and electroporated into cultured ES cells, and cultured for 10 days in G418 and gancyclovir. The surviving clones were screened with PCR, and confirmed by Southern blot after *Nco*1 digestion. Several clones were chosen and microinjected into 129Sj blastocysts prior to implantation in pseudo pregnant females. Speed congenic backcrossing into the B6 background was performed in conjunction with Dartmouse (Lebanon, NH) using high-density SNP chips, as described [37].

### Antibodies and flow cytometry

All antibodies for flow cytometry were purchased from Biolegend, eBioscience, or BD biosciences. MHC-I/peptide tetramers were synthesized and provided by the NIH tetramer core facility (Atlanta, GA). Anti-mouse CD16/32 was purchased from DartLab (Lebanon, NH). Data was acquired on a BD FACScalibur and analyzed with FlowJo software (Tree Star, Ashland, OR).

### Histopathologic evaluation of lung injury

Mice were sacrificed at the indicated time points by ketamine/xylazine anesthesia overdose followed by exsanguination according to IACUC approved protocol. Lungs were then perfused with 20 cc PBS at 25 cm H2O pressure. The tracheae were cannulated and lungs inflated with 0.5% LMP agarose. FFPE lung tissue sections were stained with hematoxylin and eosin, and the extent of lung injury was evaluated. Evaluation of pathological severity was performed in a blinded manner adapted from the methods described previously [38], [39],[40] by independently scoring of four parameters: alveolar congestion, hemorrhage, aggregation of neutrophil or leukocyte infiltration, and thickness of the alveolar wall. The area of lung sections with evidence of any of these abnormalities were measured, summed, and expressed as an percentage of the total area of the field, and a minimum of 12 fields per slide were evaluated (slides represented serial sampling of whole lung mounts separated by 10 µm levels.)

### Luminex assay and BALF albumin quantitation

Mice were sacrificed at the indicated time point and the trachea was exposed and cannulated. Bronchoalveolar lavage (BAL) was performed by flushing 1 ml of sterile PBS/HALT protease inhibitor cocktail (Thermo Pearce) back and forth 4 times. Cytospin preparations were performed on freshly diluted BAL fluid, and 30 randomly chosen high-power fields were photographed in a blinded fashion from each experimental group, and RBC were counted in each field. Remainder of collected BALF was centrifuged for 5 min. ×400 g to generate a cell free supernatant and subsequently frozen until analysis. Frozen BALF was thawed and assayed using Millipore Mouse 32-plex Luminex assay to determine the expression of cytokines and chemokines. BAL fluid albumin was analyzed by ELISA (Bethyl Laboratories, Montgomery, TX).

### Adoptive transfer

NP_366_-specific CD8^+^ T cells from WT and NKG2A^−/−^ were removed from culture 5 days after in vitro re-stimulation and expansion, and subjected to histopaque density centrifugation to remove irradiated stimulator cells. 1×10^6^ WT or NKG2A^−/−^ NP-specific cells were adoptively transferred by tail vein injection into WT mice that were intranasally infected 1 day prior with a lethal dose of Influenza PR8. Mice were monitored daily for weight change, sacrificed 5 days post infection and lungs were removed and processed for histological examination.

### Statistical analyses

Statistical analysis was performed with GraphPad Prism (GraphPad Software Inc., La Jolla, CA) using a two-tailed unpaired t-test with 95% confidence interval. Data are presented as mean ± standard deviation.

## Results

### Migration into infected tissue is required for CD8+ CD94/NKG2A expression during virus infection

**Figure 1:**
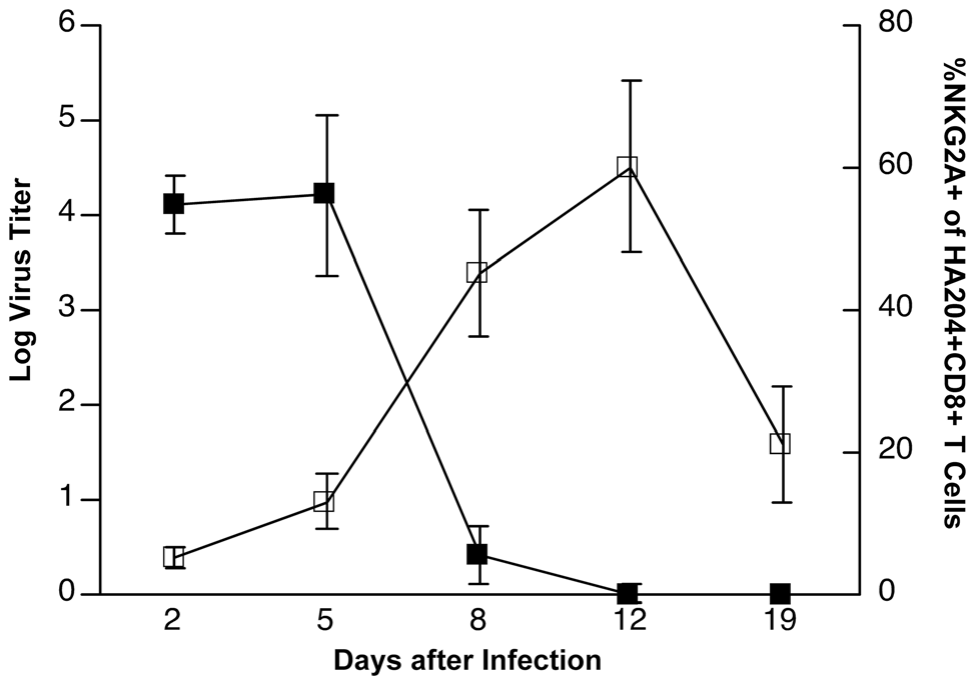
NKG2A^−/−^ expression inversely correlates to influenza viral load with peak expression at 12 days p.i. Mice were intranasally infected with a 0.5LD_50_ Influenza A/PR8/34 virus and sacrificed on the indicated days post infection. Whole lung viral titers were determined by the TCID_50_ assay, solid black squares. In parallel, lungs were harvested and NKG2A expression on NP_366_-specific CD8^+^ T cells was determined by FACS on the indicated days post- infection, open squares. Data are representative of at least 3 independent experiments.

**Figure 2:**
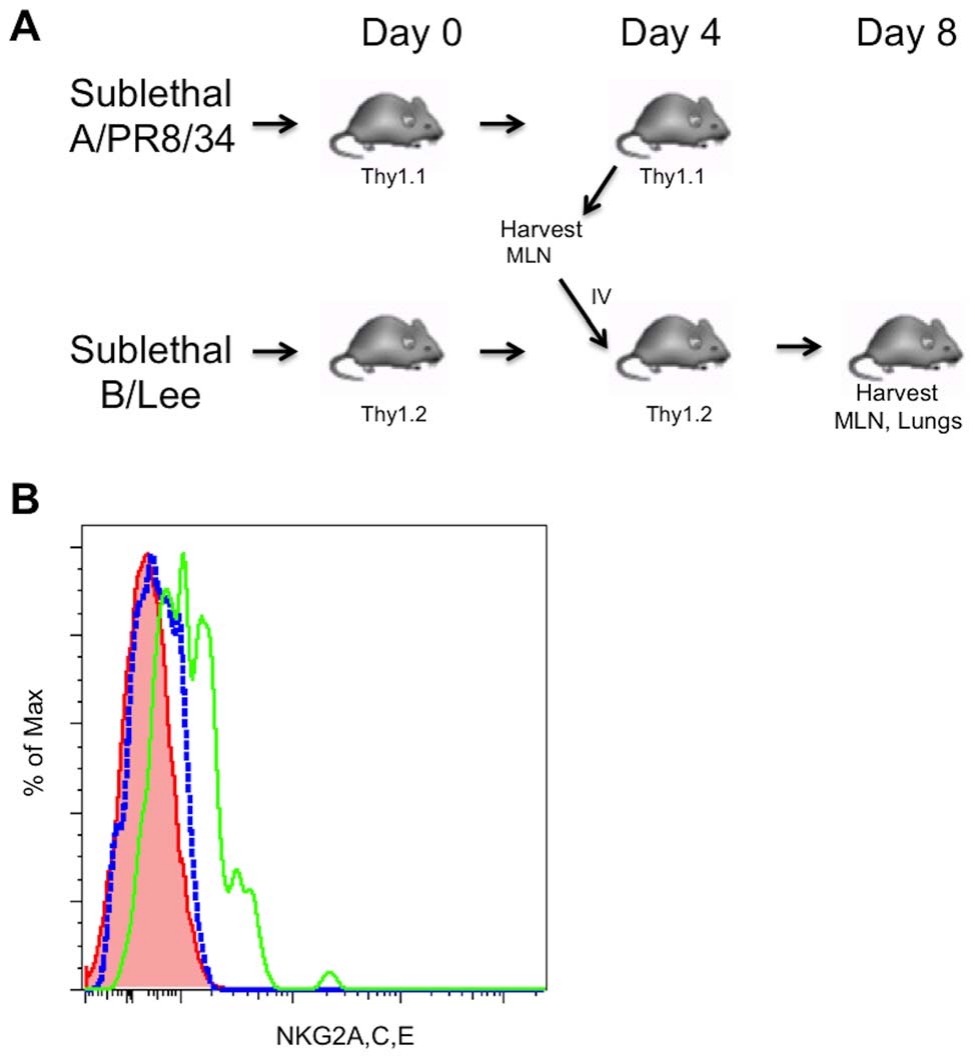
Expression of NKG2A does not require peripheral cognate antigen recognition. A. Donor Influenza A/PR8/34-specific CD8^+^ T cells isolated from Day 4 p.i. mediastinal lymph nodes were transferred into recipient mice undergoing an irrelevant Influenza B/Lee virus infection on day 4p.i. Cells were isolated from recipient mice at 8 days post influenza B/Lee infection and stained with antibodies for CD90.1 (Thy1.1), CD90.2 (Thy1.2), CD8 and NKG2A. Red area denotes NKG2A expression on recipient CD8^+^ T cells in the MLN. Blue line denotes NKG2A expression on transferred CD8^+^ T cells in the MLN. Green line denotes NKG2A expression on transferred CD8^+^ T cells in the lung. Data represents 3 mice per group pooled. Shown is a representative experiment of at least two independent experiments.

We have previously shown that the expression of NKG2A on CD8^+^ T cells activated in response to influenza infection was not observed in the MLN at any point throughout the course of infection and clearance, but that expression was observed on antigen-specific T cells harvested from the lung parenchyma at various time points [28]. The timing of expression of NKG2A on lung CD8^+^ T cells appears as virus titers were declining, suggesting the possibility that antigen load influences NKG2A expression (Figure 1). Since this raises the possibility of a relationship between antigen recognition in the lung and NKG2A expression, we tested this hypothesis with a transfer of MLN CD8^+^ T cells from A/PR8/34-infected mice into other mice sub-lethally infected with influenza B/Lee(Fig. 2a), which has no known cross-reactive T cell epitopes. As shown in figure 2b, CD8^+^ T cells harvested at day 4 after A/PR8/34 infection from the MLN showed no expression of NKG2A (histogram red area), as expected. When transferred into a B/Lee-infected mouse, the donor MLN cells harvested from the recipient MLN also show no such expression (Blue line), whereas donor cells harvested from the lung expressed NKG2A (Green line), indicating that specific antigen recognition in the lung was not a requirement for NKG2A expression. We conclude that either entry into an inflammatory milieu is sufficient to induce expression, or that exit from the suppressive milieu of the MLN is sufficient.

### NKG2A ligation *in vivo* limits tissue injury during viral clearance

**Figure 3:**
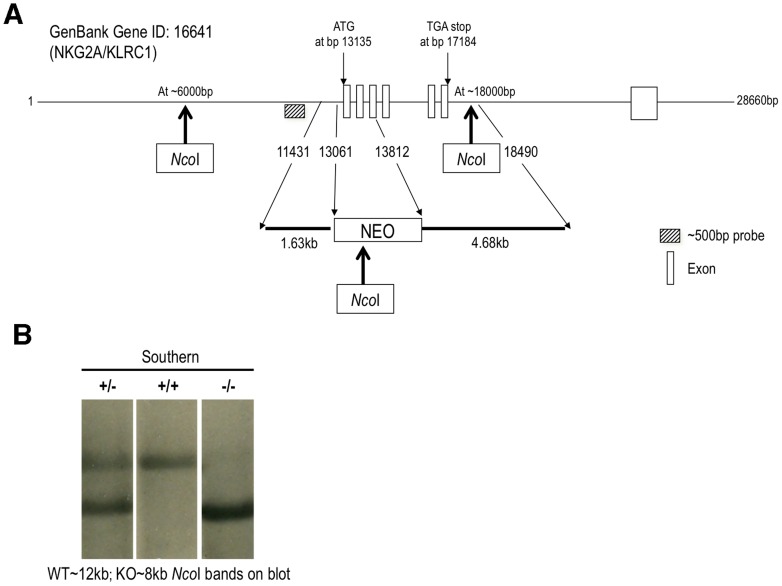
NKG2A gene knockout schema and Southern Blot. A. Schematic of the KLRC1 (NKG2A) gene showing the region of substitution of Neo cassette and the location of *NcoI* digestion sites used in generating gene fragments for southern blot for confirmation of the knockout genotype. B. Genotyping of NKG2A^−/−^ by southern blot. Lane 1 shows both WT (12 kb) and NKG2A^−/−^ (8 kb) NcoI digest fragments in heterogeneous mice, lane 2 homozygous WT (12 kb) band and lane 3 homozygous NKG2A^−/−^ (8 kb). The probe sequence is provided as Figure S1.

**Figure 4:**
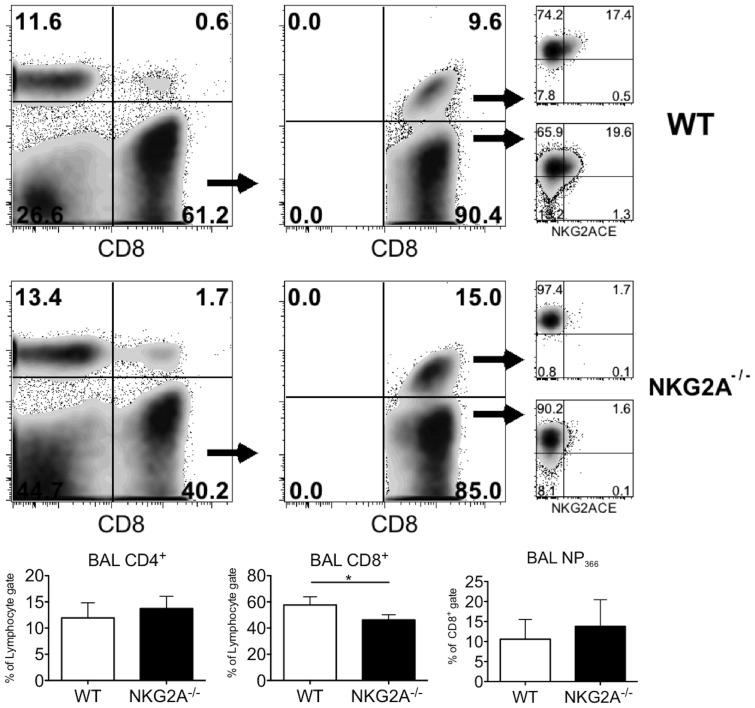
Expression of NKG2A on antigen specific CD8^+^ T cells from WT and NKG2A^−/−^ influenza infected mice. Mice were intranasally infected with a sub-lethal dose of influenza A/PR8/34 and bronchoalveolar lavage was performed on day 10 post-infection. Isolated cells were stained with NP_366_ tetramers and antibodies specific for CD44, CD8 and NKG2A,C,E or NKG2A^b6^. Surface expression was determined by FACS. Average values for CD4^+^, CD8^+^ and NP_366_ specific -CD8+ T cells from a representative experiment are shown in the bar graphs below. * p<.05 Shown is a representative experiment from at least 3 experiments with 3–4 mice per group.

Whether T cell exit from the MLN, or entry into the infected tissue, is necessary for sufficient expression, the inhibitory activity of NKG2A on CD8^+^ T cell effector function likely occurs in the lung parenchyma. The combined observation of induced expression of NKG2A on antigen specific CD8^+^ T cells in influenza infection, dampened cytokine production upon ligation of NKG2A on CD8^+^ T cells, and the dramatic immunopathology evident in Qa1-deficient mice upon CD8^+^ T cell clearance of influenza infection strongly pointed to a key role of CD8^+^ T cell NKG2A expression in abrogating tissue destruction during virus clearance [28]. In order to formally confirm this hypothesis, we generated NKG2A-null mice fully backcrossed into the B6 background (Figure 3). These mice demonstrated no defects in the numbers of immune cells in naïve mice examined at 6–8 weeks of age, indicating (as expected) that these animals exhibit normal immunological development prior to challenge (not shown). Following intranasal influenza infection, NKG2A^−/−^ mice display decreased frequency of CD8+ T cells (Fig. 4 Bottom middle) but show similar frequencies of CD4^+^ (Fig. 4 Bottom Left) and NP_366_ -specific CD8^+^ T cells (Figure 4 Bottom Right) the BAL when compared to C57BL/6 (WT) mice. WT CD8^+^ T cells exhibit induced expression of NKG2A on their surface while none was observed on NKG2A^−/−^ CD8^+^ T cells (Fig. 4 FACS, right-hand panels).

**Figure 5:**
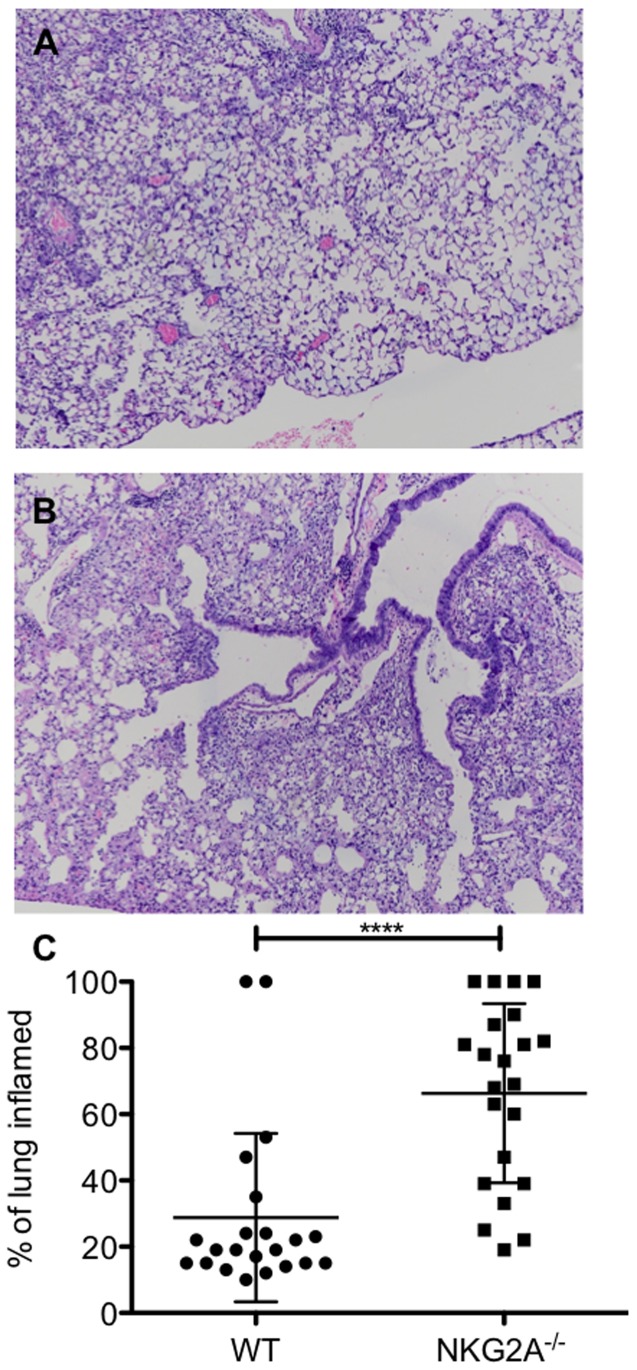
NKG2A^−/−^ mice demonstrate greater lung damage from enhanced inflammation during acute influenza infection. Mice were intranasally infected with a sub-lethal dose of influenza A/PR8/34. On day 10 post-infection, mice were euthanized by anesthesia overdose and exsanguination. Lungs were process as in materials and methods for histological sections, slides were H & E stained and evaluated for histopathological damage. Percent of total area of damaged lung per 4× field was calculated for each lung slice. Data is presented as % inflamed lung. Data is representative of two distinct experiments of 2–4 mice per group. **** p<0.0001.

As noted above, influenza infection of Qa1b^−/−^ mice followed by transfer of activated WT CD8^+^ effector cells resulted in significant lung injury compared to WT controls [28]. And while the role of Qa1b restricted CD8^+^ regulatory T cells has received considerable recent attention [41], [42], the respective role of Qa1b interaction with NKG2A expressed on effector CD8^+^ T cells has only been indirectly inferred by administration of blocking mAb to NKG2A in a non-infectious model of T cell-mediated lung injury, which resulted in enhanced immunopathology [28]. In order to confirm that the immunopathology observed in the infected Qa1b-deficient mice was in fact due to absent ligation of NKG2A, we performed intranasal infection of NKG2A^−/−^ and WT mice with 0.5 LD_50_ of influenza virus A/PR/8/34 (PR8). Animals were sacrificed 10 days post infection, and histologic analysis demonstrated that NKG2A^−/−^ (Fig. 5B) mice had significantly more inflammation in the lungs than wild-type B6 mice (Fig. 5A) and summarized in Figure 5C. Histopathological analysis demonstrated enhanced thickening of the sub-mucosa, increased consolidation, and diffuse alveolar damage. Inflammatory areas were scattered throughout all lobes of the lung in both WT and mutant mice.

### Influenza infection results in increased inflammation and inflammatory chemokine expression in NKG2A^−/−^ mice

**Figure 6:**
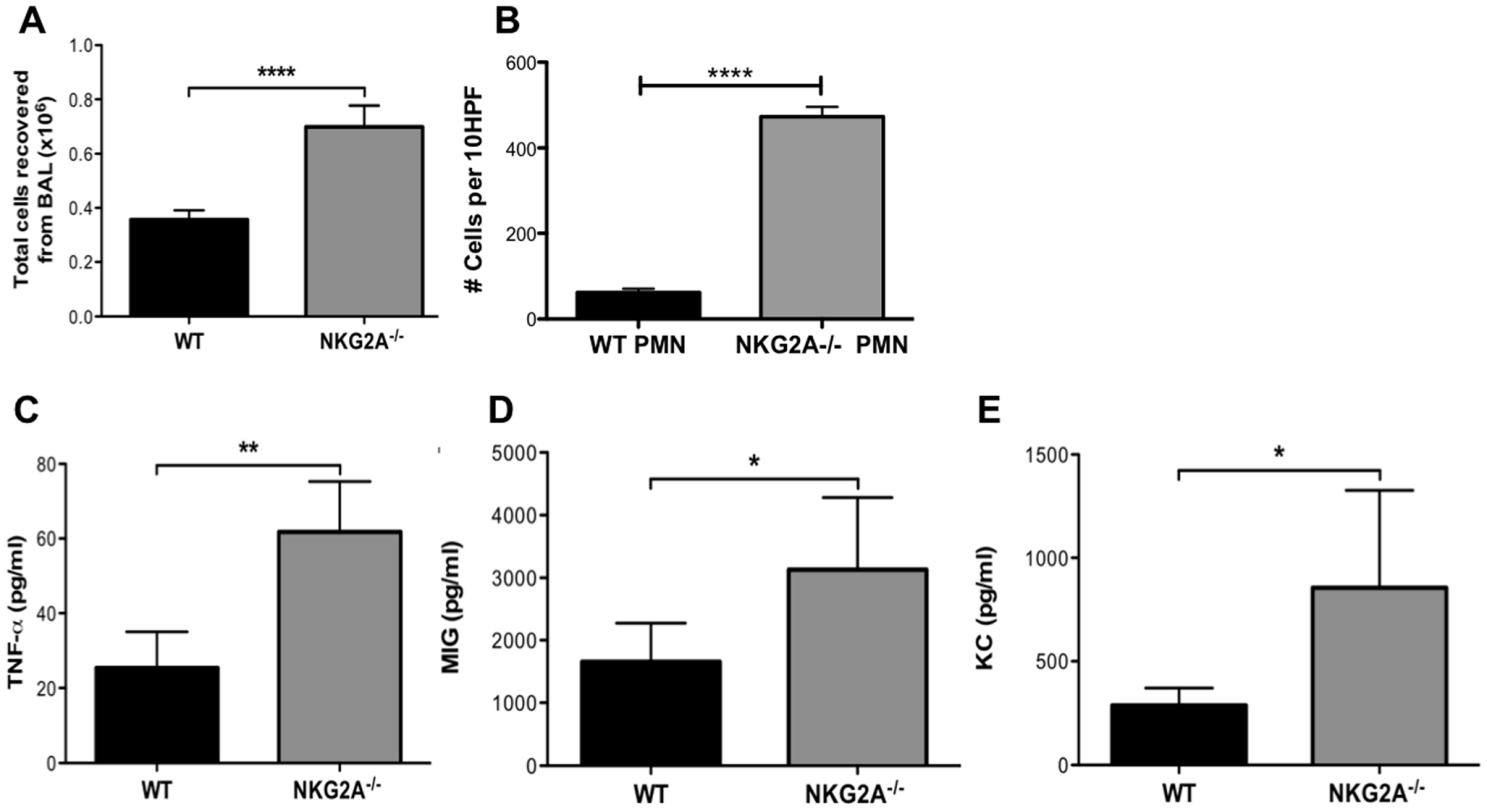
NKG2A^−/−^ mice have increased cellularity and inflammatory cytokines at 10 days p.i. Influenza PR8 infection than WT mice. Mice were intranasally infected with a sub-lethal dose of influenza A/PR8/34. On day 10 post-infection, cells were recovered from the BAL and the (A) total number of cells was enumerated. Cytospin preparations from BAL were examined for PMN (B). BALF concentrations for (C) TNF-α, (D) MIG, and (E) KC on day 10 post-infection were determined by Multiplex assay. Data are representative of two independent experiments of 5 mice per group. *p<0.05, **p<0.01, and ***p<0.005.

To further characterize the inflammatory foci observed histologically, we compared the number of cells in the lung airways, cellular distribution, and cytokine/chemokine production between C57BL/6 and NKG2A^−/−^ mice following influenza infection. At 7–8 days post infection, NKG2A^−/−^ mice had significantly greater numbers of cells in the lung airways than WT mice after collection by BAL (Fig. 6A). The increase in cell number appears to be the result of non-lymphoid cells as there was no significant increase in macrophages, CD4^+^ T cells, or CD8^+^ T cells. This increase in non-lymphoid cells was determined to be PMN as determined by analysis of BAL cytospins (Fig. 6B) Additionally, we observed significantly higher levels of TNF-α in BALF of NKG2A^−/−^ than WT (Fig. 6D), as well as increased MIG (Fig. 6E) and KC (Fig. 6F), indicating an enhanced cellular and soluble inflammatory milieu in the mutant mice than in the WT (as well as BAL fluid albumin and RBC [not shown], indicators of alveolar injury).

### Influenza specific NKG2A^−/−^ CD8^+^ T cells make increased levels of effector cytokines following peptide re-stimulation *in vitro*

**Figure 7:**
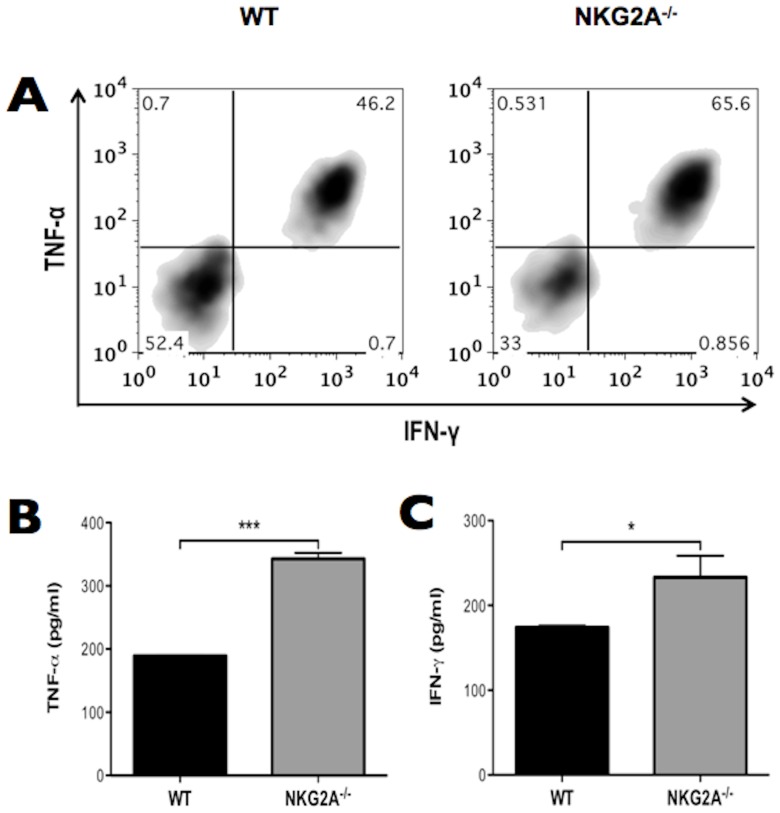
NP_366_-specific CD8^+^ T cells derived from NKG2A^−/−^ mice have enhanced effector functions. (A) NP_366_-specific WT and NKG2A^−/−^ CD8^+^ T cells were stimulated with NP_366_ peptide in the presence of brefeldin A and total production of TNF-α and IFN-γ was analyzed by FACS. Alternatively, ELISA was used to measure (B) TNF-α and (C) IFN-γ production. Data are representative of at least two experiments with 3–5 mice. *p<0.05 and ***p<0.005.

We have previously observed that blockade of NKG2A during *in vitro* stimulation of CD8^+^ T cells resulted in enhanced production of TNF-α [28]. In addition, we observed increased levels of TNF-α in the BALF at days 7–8-post influenza infection of NKG2A^−/−^ mice compared to WT. We therefore examined the *ex vivo* capacity of NKG2A^−/−^ influenza-specific CD8^+^ T cells to produce TNF-α *in-vitro* by peptide stimulation. Bulk cultures of NP_366_-specific CD8^+^ T cells were generated from the spleens of mice that had recovered from influenza infection, and cultured with or without NP_366_ peptide. As shown by representative FACS plots in figure 7A, NKG2A^−/−^ NP_366_-specific CD8^+^ T cells produced higher total levels of IFN-γ and TNF-α than did NP_366_-specific cells from WT mice after *in vitro* peptide stimulation. The enhanced release of TNF-α (Fig. 7B) and IFN-γ (Fig. 7C) after *in vitro* stimulation by NKG2A^−/−^ mice was further quantified by ELISA. NKG2A^−/−^ CD8^+^ T cells also produced higher levels of IL-2 than did WT upon stimulation (not shown). This indicates that antigen-specific CD8^+^ T cells from NKG2A^−/−^ mice express greater effector activities upon stimulation than WT, confirming our observations using antibody blockade [28].

### Infected WT recipients of transferred NKG2A^−/−^ CD8^+^ T cells exhibit increased inflammation and enhanced alveolar hemorrhage following lethal influenza challenge

**Figure 8:**
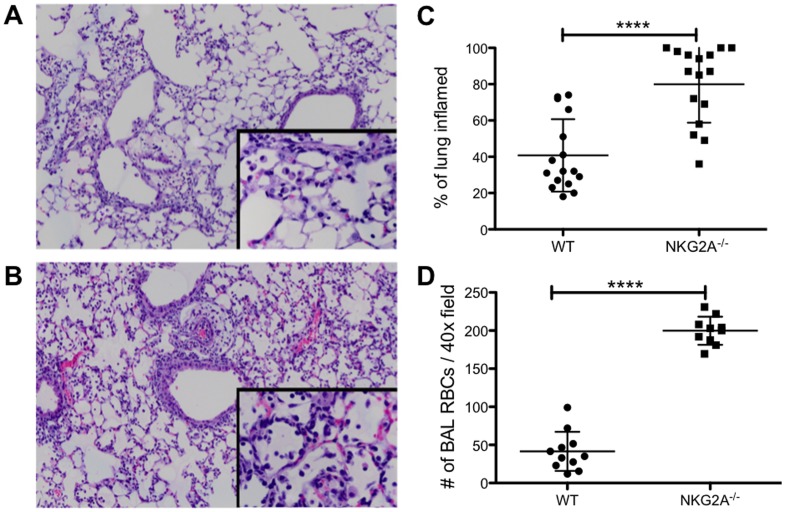
Transferred NKG2A^−/−^ CD8^+^ T cells are more inflammatory following intranasal influenza PR8 infection than transferred WT CD8^+^ T cells. Whole lung histology on day 5 post-infection from mice receiving either (A) WT or (B) NKG2A^−/−^ NP_366_-specific CD8^+^ T cells after lethal challenge with influenza A/PR8/34 virus. Shown are representative slides from at least 2 separate experiments at 10× and 40×(inset) with overall histolopathological score was determined as described in Materials and Methods(C). Hemorrhage after T cell transfer was enumerated by counting the number of RBCs from multiple 40× fields of tissues from multiple mice (D). Data are representative of the results of two experiments with 3–5 recipient mice per group. *p<0.0001.

The results shown above strongly suggest that the enhanced immunopathology observed in influenza-infected NKG2A^−/−^ mice was a result of the absence of the inhibitory signal to antigen-specific CD8^+^ T cells. However, NKG2A is expressed on NK cells as well, and though NK cells appear to play no significant role in influenza virus clearance or immunoregulation in influenza, we endeavored to formally exclude the potential contribution of NKG2A-deficiency on the NK cells to the observed immunopathologic phenotype. We therefore performed adoptive transfer of activated NKG2A^−/−^ CD8^+^ T cells (versus WT) into influenza-infected WT mice. Bulk cultures of NP_366_-specific CD8^+^ T cells generated from NKG2A^−/−^ or WT mice were stimulated *in vitro* and intravenously transferred to WT mice infected with a lethal dose of influenza on the day prior to transfer. Mice were sacrificed 4 days after transfer (5 days post infection). WT mice receiving NKG2A^−/−^ cells exhibited greater weight loss than recipients of WT T cells following influenza infection (not shown). Histopathological analysis confirmed that mice receiving NKG2A^−/−^ CD8^+^ T cells had more inflammatory foci (Fig. 8B and 8C) than did WT mice receiving WT CD8^+^ T cells (Fig. 8A and 8C). Importantly, beyond increased inflammation, alveolar hemorrhage was dramatically enhanced in infected recipients of NKG2A−/− CD8^+^ T cells compared to WT (figure 8D), indicating that absence of NKG2A only on effector CD8^+^ T cells is sufficient to drive enhanced lung injury.

### Adenovirus-infected NKG2A^−/−^ mice have enhanced liver damage compared to wild-type mice

**Figure 9:**
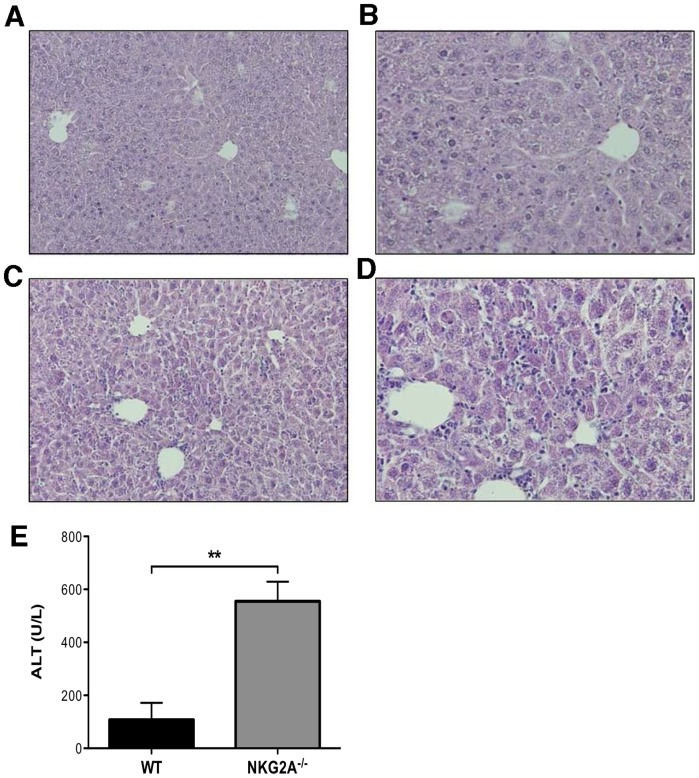
NKG2A^−/−^ mice have greater liver inflammation and enhanced cellular damage following Adenovirus infection than WT mice. H & E stained histological sections of liver from WT mice at (A) 10× and (B) 40× or NKG2A^−/−^ mice at (C) 10× and (D) 40×7 days post Adv infection. Serum alanine transaminase (ALT) levels 7 days post Adv infection. Data are representative of two independent experiments of three mice per group. *p<0.01.

To examine whether regulation of effector CD8^+^ T cell function by NKG2A extended beyond influenza infection, we examined the consequence of NKG2A-deficiency during an acute infection with murine adenovirus (Ad-OVA). Histological sections from livers of WT and NKG2A^−/−^ mice infected seven days previously with Ad-OVA were examined and we observed increased lymphocytic infiltrate in the livers of NKG2A^−/−^ mice (Figures 9C, 9D) compared to WT (Figures 9A, 9B). As a measure of liver injury, serum alanine aminotransferase (ALT) was measured and we observed an increase in serum ALT levels in NKG2A^−/−^ mice compared to WT mice, 7 days after Ad-OVA infection (Figure 9E). We also examined cytokine levels at day 7 post Ad-OVA infection, and NKG2A^−/−^ mice had significantly higher message levels for IFN-γ than WT mice. This corresponds to the increase in the absolute numbers of liver CD8^+^ T cells at this time point (data not shown). In contrast to our observations in influenza infection, the TNF-α mRNA levels after Ad-OVA infection were not significantly different. There was also no difference in Ad-OVA virus clearance between mutant and WT mice, confirmed by qPCR of OVA mRNA (not shown). Thus, similar to influenza virus infection, the lack of NKG2A resulted in enhanced liver injury in adenovirus hepatitis.

## Discussion

Virus infection frequently results in tissue injury as a consequence of both the cytopathic effects of the virus as well as the damage induced by immune-mediated virus clearance, whether successful or not [15], [20], [43]–[45]. It has become increasingly evident that the contribution of immune responses, both innate and adaptive, to tissue injury may be much more significant than previously appreciated. Greater understanding of the pathogenesis of multiple virus infections has led to discovery of multiple control mechanisms that have evolved to limit the immunopathologic impact on tissue structural and functional integrity. This appears to be advantageous to the host even when virus clearance is unsuccessful, such as in polyoma virus infection, LCMV, HIV, and hepatitis [25], [46]–[48]. The mechanisms of both tissue injury and maintenance of integrity largely overlap with antiviral effector functions, such as type I interferon, which is both antiviral and anti-inflammatory [49]. Effector activities of both innate and adaptive immune cells, including production of IFN-γ and TNF-α, which are both antiviral and pro-inflammatory, but also the anti-inflammatory cytokine, IL-10 [21], are subject to counter regulation. This counter regulation presumably serves to limit injury and allow maintenance of tissue integrity and function, a balance, which is critical to the survival of the infected host. Counter regulatory, i.e. inhibitory receptors, for innate cells such as NK cells are well described. Several inhibitor molecules on CD8^+^ T cells have been also reported which are expressed at various time points after infection, such as CTLA-4, PD-1, TIM-3, among others [22].

In this study, we demonstrated that NKG2A, an inhibitory receptor normally associated with NK cells, negatively controls the function of effector CD8^+^ T cells and the extent of injury during two different viral infections, influenza virus and adenovirus. Expression of NKG2A on CD8^+^ T cells occurred exclusively in the lung late during influenza infection (i.e. day 8), when virus titers were falling, suggesting an important role for NKG2A in resolution of the effector phase. In support of this hypothesis, NKG2A ligation with cognate receptor functioned to limit CD8^+^ T cell effector functions, as CD8^+^ T cell production of effector cytokines, TNF-α and IFN-γ, was enhanced in the absence of NKG2A. Enhanced cytokine production resulted in exacerbated lung and liver injury during influenza virus and adenovirus infection, respectively. Importantly, complete absence of NKG2A or absence of NKG2A only on effector CD8^+^ T cells did not impair viral clearance. Taken together, these observations identify NKG2A as a critical negative regulator of CD8^+^ T cells, limiting effector function and the extent of injury during viral infection, while permitting successful clearance of infection.

Our data suggests that the exacerbated tissue injury observed during both adenovirus and influenza virus infection was largely due to enhanced CD8^+^ T cell effector functions. During adenovirus infection, enhanced hepatocellular injury observed in the absence of NKG2A correlated with an increase in the absolute numbers of CD8^+^ T cells and IFN-γ expression in the liver. This is consistent with other models of liver infection where the onset of hepatocellular injury correlates with the acquisition effector functions of CD8^+^ T cells [50]. In the context of influenza infection, despite no changes in the absolute numbers of CD8^+^ T cells in the airways in the absence of NKG2A, we observed increased levels of effector CD8^+^ T cell cytokines, such as TNF-α. This was presumably due to increased production of TNF-α by influenza-specific CD8^+^ T cells as NKG2A-deficiency resulted in enhanced production of TNF-α and IFN-γ by these cells *in vitro*. We have previously shown that TNF-α production by influenza-specific CD8^+^ T cells is critical for the development of severe and lethal lung injury during influenza infection [28]. The mechanisms by which expression of TNF-α by influenza-specific CD8^+^ T cells mediate this lethal injury are through the induction of chemokines by alveolar epithelial cells and the subsequent recruitment of inflammatory cells into the lungs [28]. Consistent with our previous observations, we observed enhanced levels of chemokines, such as MIG and KC, in the absence of NKG2A and subsequently, increased numbers of inflammatory cells in the airways and exacerbated lung injury. Absence of NKG2A only on influenza-specific CD8^+^ T cells also resulted in exacerbated lung injury, indicating that NKG2A primarily functioned by regulating the extent of effector CD8^+^ T cell-mediated immunopathology. Interestingly, increased levels of TNF-α, MIG, and KC have been observed in models of highly pathogenic influenza infection [26], [51], [52], suggesting that failure to properly regulate expression of NKG2A on effector cells may contribute in part to enhanced expression of pro-inflammatory mediators and exacerbated pulmonary pathology during highly pathogenic influenza infection.

These observations raise the critical question of what are the mechanisms that regulate NKG2A expression on effector CD8^+^ T cells during virus infection. We have previously shown that NKG2A expression is absent on naive CD8^+^ T cells, but was induced in response to influenza infection [28]. Interestingly, we show here that the expression of NKG2A occurs only in the periphery and only when virus titers (and antigen load) were waning. This is somewhat curious, since we found that specific antigen in the periphery was unnecessary for expression. The nature of the signal(s) which lead to CD8^+^ NKG2A expression have not been clearly identified. While various *in vitro* studies have suggested that IL-15 or IL-10 may play a role in induction of CD8^+^ NKG2A expression [26], [52], [53], we were unable to demonstrate any such effect of these cytokines in vivo (not shown). One tantalizing *in vitro* study suggested that high levels of IL-2 (and presence of CD28) inhibited expression of NKG2A on CD8^+^ T cells [54], which raises the interesting possibility that higher IL-2 levels in the MLN than in the periphery may account for the anatomical predilection for NKG2A expression in the lung. Thus, as with some of the other aforementioned molecules that dampen or suppress effector T cell responses with a variety of other mechanisms, NKG2A on CD8^+^ T cells appears to modulate effector cytokine and chemokine production allowing a transition to recovery. While there are many redundancies of inhibition pathways, we have shown that deletion of NKG2A on CD8^+^ T cells can have a direct and non-redundant detrimental effect resulting in increased immunopathology following virus infections.

In summary, we have shown that antiviral CD8^+^ T cells have an additional control mechanism in NKG2A expression, which we hypothesize allows for fine control of effector function in the context of virus clearance. We propose that this, and other such mechanisms, are critically important regulators which fine-tune CD8^+^ T cell effector functions, serving to clear virus from physiologically critical tissues while minimizing tissue injury and allowing maintenance of structural and functional integrity so important to survival of the host.

## Supporting Information

Figure S1 **Probe sequence (562 bp) for detecting NKG2A knockout, heterozygous, and WT mice by Southern blot, consisting of** ***NcoI*** **-digested tail-tip DNA.** Underlined nucleotides indicate Forward and Reverse primers, respectively, used to amplify the probe with digoxygenin-labeled UTP from a plasmid containing a portion the NKG2A gene in the Balb/c mouse. This probe sequence corresponds to the range of 131393104 to 131393665 of Mm_Celera alternate assembly on mouse chromosome 6, accession number AC_000028.1.
(DOCX)
