# Supplementary material for: Tissue-Protective Effects of NKG2A in Immune-Mediated Clearance of Virus Infection
Source: PLoS One. 2014 Sep 24;9(9):e108385. doi: 10.1371/journal.pone.0108385 (PMC4177548; doi:10.1371/journal.pone.0108385)
Supplement: Figure S1 — Probe sequence (562 bp) for detecting NKG2A knockout, heterozygous, and WT mice by Southern blot, consisting of NcoI -digested tail-tip DNA. Underlined nucleotides indicate Forward and Reverse primers, respectively, used to amplify the probe with digoxygenin-labeled UTP from a plasmid containing a portion the NKG2A gene in the Balb/c mouse. This probe sequence corresponds to the range of 131393104 to 131393665 of Mm_Celera alternate assembly on mouse chromosome 6, accession number AC_000028.1. (DOCX) [file pone.0108385.s001.docx]

TTTGCTTGCTTGCTTGCTTACTTGCTTGTTTTGACAGAACTGTGCTGCTTCTTGCCAGTGTACCTATTGCACATCCAGCCACAAACCCTCTAACTTTTCTGTATTCCCAACCTCTTCATTTTAACCAATAAAACTGTGTTACCTACTCCTTCTTTATGATGTTATTTTTAAAGGCACTTTTTTCAGTTTCTGATTTCTAAATTAAAATGCTACTGAGTTTTTATTCAATCCCTACATTTTAAAAACTGAATTTGCTTATTTCCTTCTAAGTGTTTTGTTACTGTGTTTAGGCTTCCATGCCAAGATTATGTAATGTCAAAAATAGGTGATTTAATTTAAATCCTCCTGATAGTGACAGTTTTGTCAGAGAATCCTATAAATTACTATTTTTAATTATGAAATAAAACATAAGGGTGTTGGGAACATATCTTAATTGTTAAGAGCACACTCTGATCATCCAGAGGTTTCATGTTCAATCCCCAGGACCCACATGGTGGTATACATTCTTCTATAGCTCCAGTTTAAGGCGATCCAATGCCATTTTCTGCCCTCCAAGAACACT
